# Radiomics-Based Prediction of TERT Promotor Mutations in Intracranial High-Grade Meningiomas

**DOI:** 10.3390/cancers15174415

**Published:** 2023-09-04

**Authors:** Burak Han Akkurt, Dorothee Cäcilia Spille, Susanne Peetz-Dienhart, Nora Maren Kiolbassa, Christian Mawrin, Manfred Musigmann, Walter Leonhard Heindel, Werner Paulus, Walter Stummer, Manoj Mannil, Benjamin Brokinkel

**Affiliations:** 1 Department of Radiology, University Hospital Muenster, DE-48149 Muenster, Germanym.mannil@bbtgruppe.de (M.M.); 2 Department of Neurosurgery, University Hospital Muenster, DE-48149 Muenster, Germany; 3 Institute of Neuropathology, University Hospital Muenster, DE-48149 Muenster, Germanywerner.paulus@ukmuenster.de (W.P.); 4 Department of Neuropathology, University Hospital Magdeburg, 39120 Magdeburg, Germany; 5 Institute for Diagnostic and Interventional Radiology, Caritas-Hospital, DE-97980 Bad Mergentheim, Germany

**Keywords:** human telomerase reverse transcriptase, magnetic resonance imaging, meningiomas, radiomics, TERT

## Abstract

**Simple Summary:**

Mutations in the telomerase reverse transcriptase (TERT) gene promoter are rare in meningiomas, but are associated with more aggressive forms of meningiomas as well as recurrence and progression. In this study, we analyzed data of patients who underwent meningioma surgery with neuropathologically assessed TERT promoter mutation status. Semiautomatic segmentation of the meningiomas was performed on preoperative magnetic resonance imaging (MRI). Radiomics features were extracted from T1-weighted contrast-enhanced MRI data. TERT promoter mutations were predicted using the random forest algorithm with an increasing number of radiomic features. 117 image sets were included and divided into a training group with 94 image sets and a test group with 23 image sets. Based on the test group, established five- and eight-feature models demonstrated high sensitivity, specificity, accuracy and AUC values for prediction of TERT promoter mutation status.

**Abstract:**

Purpose: In meningiomas, TERT promotor mutations are rare but qualify the diagnosis of anaplasia, directly impacting adjuvant therapy. Effective screening for patients at risk for promotor mutations could enable more targeted molecular analyses and improve diagnosis and treatment. Methods: Semiautomatic segmentation of intracranial grade 2/3 meningiomas was performed on preoperative magnetic resonance imaging. Discriminatory power to predict TERT promoter mutations was analyzed using a random forest algorithm with an increasing number of radiomic features. Two final models with five and eight features with both fixed and differing radiomics features were developed and adjusted to eliminate random effects and to avoid overfitting. Results: A total of 117 image sets including training (*N* = 94) and test data (*N* = 23) were analyzed. To eliminate random effects and demonstrate the robustness of our approach, data partitioning and subsequent model development and testing were repeated a total of 100 times (each time with repartitioned training and independent test data). The established five- and eight-feature models with both fixed and different radiomics features enabled the prediction of TERT with similar but excellent performance. The five-feature (different/fixed) model predicted TERT promotor mutation status with a mean AUC of 91.8%/94.3%, mean accuracy of 85.5%/88.9%, mean sensitivity of 88.6%/91.4%, mean specificity of 83.2%/87.0%, and a mean Cohen’s Kappa of 71.0%/77.7%. The eight-feature (different/fixed) model predicted TERT promotor mutation status with a mean AUC of 92.7%/94.6%, mean accuracy of 87.3%/88.9%, mean sensitivity of 89.6%/90.6%, mean specificity of 85.5%/87.5%, and a mean Cohen’s Kappa of 74.4%/77.6%. Of note, the addition of further features of up to *N* = 8 only slightly increased the performance. Conclusions: Radiomics-based machine learning enables prediction of TERT promotor mutation status in meningiomas with excellent discriminatory performance. Future analyses in larger cohorts should include grade 1 lesions as well as additional molecular alterations.

## 1. Introduction

In meningiomas, mutations of the Telomerase Reverse Transcriptase (TERT) promotor have been detected in a small subset (<5–15%) of tumors [1,2] with considerable worse prognosis [3]. Correspondingly, the detection of a TERT promotor mutation now qualifies the diagnosis of anaplasia [4] according to the current fifth edition of the WHO Classification of Central Nervous System Tumours [4], with potential subsequent impact on postoperative treatment. Given their low frequency, a generalized screening of all meningiomas for TERT promoter mutations is considered inefficient, and the current WHO classification of brain tumors recommends targeted sampling of specimen displaying grade 2/3 borderline histological characteristics and in tumors with aggressive biological behavior [4]. Identification of risk factors associated with TERT promotor mutations in meningiomas are urgently needed to enable systematic screening in selected patients.

Radiomics is an emerging field that compromises quantitative analyses of features in predefined regions of interest (ROIs) on radiological imaging and their correlations with, e.g., histological data or tissue characteristics, genetic and molecular alterations, or course of the disease in both central nervous [5] and extra-neural neoplasms [6]. Radiomics performed on magnetic resonance imaging (MRI) were shown to predict molecular alterations such as IDH1/2 mutations [7,8], 1p19q co-deletion [9], ATRX status [10], MGMT promoter methylation [11] and also TERT promoter mutations [12] in gliomas with remarkable accuracy. In meningiomas, numerous studies analyzed correlations of radiomics features with intraoperative and histological characteristics or prognosis [13,14], while the predictive value for molecular alterations has not been investigated yet. Of note, conventional MRI analyses revealed distinct anatomical distributions of key genetic and epigenetic alterations such as DNA methylation [15] or hotspot mutations including TERT [16,17,18,19] as well as their correlations with further imaging characteristics [16,18,20], indicating the immense potential of imaging data to predict molecular alterations in meningiomas.

Considering their impact on meningioma grading and therapy, their low frequency, and the corresponding need for targeted screening analyses, we therefore analyzed the value of radiomics to predict TERT promoter mutations in series of intracranial high-grade meningiomas.

## 2. Methods

### 2.1. Data Recovery and TERT Sequencing

Archives of the Institute of Neuropathology of the University Hospital Muenster were screened for all histopathologically confirmed grade 2/3 meningiomas operated in the local Department of Neurosurgery between 1991 and 2019. All cases with eligible preoperative MRI and DNA in sufficient quality/amount ratios were included. Clinical information was obtained from pathology reports and preoperative imaging and included patient age and sex at the date of surgery, as well as the tumor location (classified into convexity, parasagittal, skull base and others). Diagnoses and grading were performed for former studies [21,22,23] according to the 2016 Classification of Central Nervous System Tumours in all cases. Correlations between conventional imaging characteristics and TERT promotor mutations were previously investigated in a subset of the present cohort [19]. C228T and C250T TERT promoter mutations were determined by Sanger sequencing as described previously [19,24]. Briefly, genomic DNA was isolated from formalin-fixed paraffin-embedded meningioma specimen (FFPE Plus LEV DNA Purification Kit, Promega GmbH, Walldorf, Germany), quantified, cleaned (OneStep PCR Inhibitor Removal Kit, Zymo Research Europe GmbH, Freiburg, Germany) and subjected to multi-step polymerase chain reaction (PCR) using the following primer pairs: forward: 5′-GCG CTG CCT GAA ACT CGC-3′; reverse: 5′-CGT CCT GCC CCT TCA CCT-3′, Eurofins Genomics, Ebersfeld, Germany [19,24]. For both primers, subsequent sequencing PCR was performed using Big Dye Terminator v3.1 Cycle Sequencing via an automatic DNA sequencer (ABI PRISM 3130 Genetic Analyzer, Thermo Fisher Scientific, Waltham, MA, USA). PCR products were quantified, and fragments were visualized and analyzed according to the predefined fragment-size PCR products by gel electrophoresis using the Agilent 2200 Tape Station (Agilent Technologies, Santa Clara, CA, USA). Final DNA sequences were analyzed by electropherograms using standard software (Sequence Scanner V1.0, Applied Biosystems, Thermo Fisher Scientific, Waltham, MA, USA).

### 2.2. Radiomics

**Figure 1:**
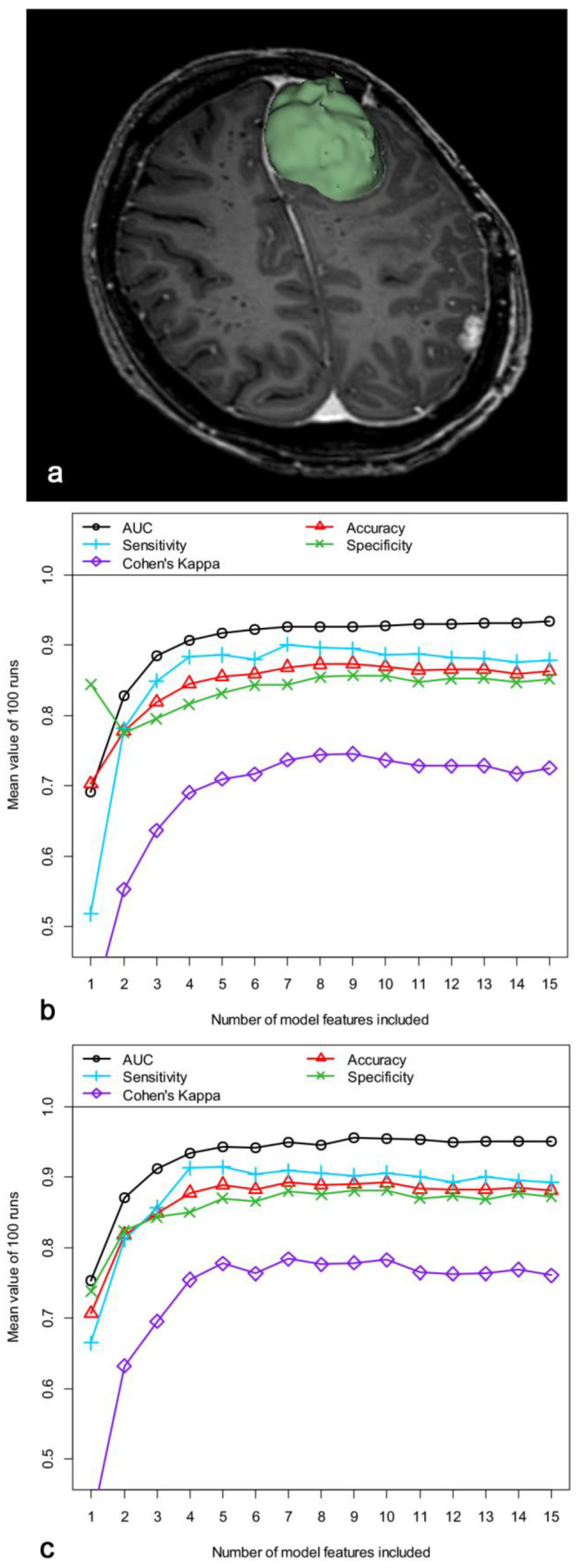
Illustrative sample of semiautomatic tumor segmentation and prediction of TERT promotor mutation status in meningiomas. (**a**) Semiautomatic segmentation of T1-weighted, magnetic resonance imaging of a parasagittal left frontal meningioma. Area under the curve (AUC), accuracy, sensitivity, specificity, and Cohen’s Kappa for the independent test samples, calculated as means of 100 repetitions (100 cycles) depending on the number of model features included. Feature pre-selection and model construction were performed using random forest algorithm (**b**). In (**c**), the features were fixed and included into the models in order of importance. Subsequent model development was again performed using the random forest algorithm.

Radiomics features were analyzed in preoperative T1-weighted contrast-enhanced MR images of 67 patients with available imaging and TERT promotor mutation status (see illustrative Figure 1a). All images were obtained on 1.5 and 3 T MR scanners of Philips Healthcare (Amsterdam, The Netherlands). Due to the rarity of mutations, patients with mutations were segmented multiple times as a data augmentation technique for the training of our proposed model, leading to a total of 117 image datasets, of which 51 images were assigned to patients with and 66 images to patients without TERT promoter mutations (Supplementary Table S1). Segmentation of the meningiomas was semi-automatically performed using the 3D Slicer open-source software platform (version 4.10, www.slicer.org (accessed on 08 December 2022)) and utilizing Segmentation Wizard. Subsequently, a total of 107 radiomic features were determined using the open-source package PyRadiomics, which is an implementable plugin for the 3D slicer platform. These radiomic features extracted from the regions of interest (ROI) belong to different feature classes: (1) 18 first-order statistics features, (2) 14 shape-based features, (3) 24 gray level co-occurrence matrix features, (4) 16 gray level run length matrix features, (5) 16 gray level size zone matrix features, (6) 5 neighboring gray tone difference matrix features, and (7) 14 gray level dependence matrix features. All features were z-score transformed and subjected to a 95% correlation filter to exclude redundant information.

### 2.3. Statistical Analysis

Statistical analysis was performed using the R software (version 4.1.2). The 117 images used to predict TERT promotor mutation status were randomly assigned to groups of training (80% of the data, *N* = 94) and independent test data (the remaining 20% of the data, *N* = 23), i.e., we used a stratified 4:1 ratio with a balanced distribution of TERT-mutated and non-mutated patients between the two groups (compare Supplementary Table S1). The training data were used solely for the feature pre-selection and the subsequent model development. A random forest algorithm was used for model development. Hyperparameters included in the models were optimized using 10-fold cross-validation. The discriminatory power of the models was subsequently determined using the independent test data.

The optimal number of features was determined during model development by including an increasing number of features (starting with a single-feature model). The importance of the features was based on the training data using “varImp”—a function in R (varImp = variable importance). For each model with a predefined number of features, the most important features were included in these models. The optimal number of model features was defined by determining the highest model performance with respect to the independent test data to minimize the risk of overfitting. According to previous descriptions [25], data partitioning and subsequent model development were repeated a total of 100 times (each time with newly partitioned training and independent test data) to eliminate the random effects and to demonstrate the robustness of the approach. Two final models were subsequently established containing, respectively, 5 and 8 features that were most frequently selected. Using these fixed features, the data partitioning, the training of the models and the subsequent determination of the performance with independent test data were also repeated 100 times in order to also be able to determine the robustness of the results obtained with the fixed model. Performance values were finally calculated as averages of 100 cycles (i.e., using 100 different data splits for training and testing) based on the independent test data. To analyze the potential statistical influence of multiple segmentations, we finally compared the discriminatory power of each included feature by single segmentation of only one randomly selected set of each patient. Mean discriminatory power was calculated after 10-fold repetition to minimize random effects. AUC, accuracy, sensitivity, specificity and Cohen’s Kappa were calculated to quantify the discriminatory power of our models. Data collection and scientific use were approved by the local ethics committee (Münster 2018-061-f-S).

## 3. Results

Supplementary Tables S1 and S2 summarize baseline clinical data and TERT promotor mutation status of the included patients as well as the TERT promotor mutation status of the training and independent test data. In general, the modelling approach predicted TERT promoter mutations with remarkable discriminatory power. Figure 1b,c show the AUC, accuracy, sensitivity, specificity, and Cohen’s Kappa obtained with our modelling approach as a function of the number of features included in the models. Of note, a significant increase in the average discriminatory power up to the fifth feature included was observed, while the addition of further features (up to the seven- or eight-feature model) only achieved a slight further increase. At higher model complexity, the discriminatory power slightly decreased, indicating the beginning of overfitting when more than eight features were included.

The analysis of the features selected in the 100 runs revealed a very similar feature composition. Table S2 shows the number of runs in which the individual features were selected for the models with eight included features in the total of 100 runs. The two features, “MCC” and “Location = falx (yes/no)”, were selected almost without exception during the 100 runs. The next three most important features, “DependenceEntropy”, “LargeDependenceHigh GrayLevelEmphasis” and “Minimum”, were selected in at least 3/4 of the runs, and features “RunEntropy”, “SizeZoneNonUniformity” and “GrayLevelNonUniformity.2” were selected in at least 2/3 of the runs.

Repeated model development with different data partitions and fixed model features confirmed the most achievable discriminatory power for the detection of TERT promoter mutations including the first 5–8 features (Figure 1b,c). Table S3 summarizes the benchmark values for the discriminatory power for predicting TERT mutation status of the five- and eight-feature models, with a slightly better performance of the eight-feature model (different features/fixed features): mean AUC = 92.7%/94.6%, mean accuracy = 87.3%/88.9%, mean sensitivity = 89.6%/90.6%, mean specificity = 85.5%/87.5%, and mean Cohen’s Kappa = 74.4%/77.6%.

Single segmentation from each patient revealed similar AUC values compared to multiple segmentations, confirming the feasibility of our approach and the good discriminatory power of radiomics to predict TERT promotor mutations (Supplementary Table S3).

## 4. Discussion

Numerous studies reported correlations of tumor and peritumoral characteristics on routine preoperative imaging, such as lesion or edema volume and contrast enhancement, with both the WHO grade and prognosis in meningioma patients. Notably, imaging characteristics correlated with the WHO grade as determined by histopathology, but they are not necessarily congruent with those related with progression [26], eventually indicating the influence of prognostic molecular alterations not considered for grading in the analyzed studies.

Along with the increasing knowledge about key molecular alterations in meningiomas, correlations, e.g., of NF2, TRAF7, KLF4, PIK3CA, SMO, SMARCB1, POLR2A, SUFU, and POLR2A mutations or DNA methylation patterns with characteristics on routine preoperative imaging have largely been described [15,16,17,18,20,27,28]. Of note, the latter are restricted to tumor location and the peritumoral edema and investigator-based conventional imaging analyzes. In our own series, we further demonstrated a para-sagittal tumor location as a strong predictor for TERT promotor mutations in high-grade meningiomas [19]. Similarly, three-dimensional fractal dimension and lacunarity features were previously shown to strongly correlate with TERT promotor mutation status [29], further underlining the immense potential of imaging characteristics to predict molecular alterations in general, and especially of TERT, in meningiomas. However, investigator-based imaging analyses typically suffer from subjectivity and subsequent inter-observer variability and are mostly restricted to small patient cohorts. Radiomics-based imaging analyses are considered to overcome these shortcomings and enable (semi-)automatic testing in large patient series [30].

In contrast to gliomas, in which associations between radiomics features and molecular alterations have largely been investigated [7,9,11,12], corresponding data on meningiomas are lacking to date. Hence, as the first study so far, we here demonstrate the capability of radiomics to predict TERT promotor mutations in high-grade meningiomas in routine preoperative imaging, with considerable reliability according to Cohen’s Kappa analyses. Models were developed in repetitive 100-fold training and test data sets and similar performance was gained comparing different methods of data analysis, hence indicating the robustness of the gained results.

Given the low frequency of TERT promotor mutations in meningiomas in general and in grade 1 lesions in particular, the current WHO classification recommends “consideration (of TERT sequencing) in clinically aggressive atypical meningiomas or those with borderline CNS WHO grade 2/3 features” [4]. In fact, the detection of promotor mutations directly impacts grading, decision regarding adjuvant irradiation therapy and eventually study inclusion. Spiegl-Kreinecker et al. further reported sensitivity of TERT promotor mutated meningiomas to the E-twenty-six (ETS) transcription factor inhibitor YK-4–279, indicating TERT promotor mutations also as a potential predictive marker for targeted therapies [31]. Thus, current clinical relevance of an effective and exact screening for TERT promotor mutation is severe and will likely further increase. In this context, radiomics might improve the effectiveness of identification of patients at risk for TERT promotor mutations and enable more specific future molecular screening, allowing for improvement of diagnostics and eventually treatment. Aside from application during standard clinical routine, radiomics-based screening eventually allows (semi-) automatic analyses, e.g., of previous retrospective larger patient series in which genetic alterations have formerly not been considered. Our results further encourage future analyses of the predictive value of radiomics in meningiomas for other molecular alterations, e.g., methylation classes or homozygous CDKN2A/B deletions. On the other hand, imaging-based replacement of laboratory analyses and qualified interpretation of the results is currently not foreseeable.

The authors are aware of some limitations of the study. Although providing analyzes in a considerable cohort size of high-grade meningiomas, our study suffers from its retrospective nature and limitations due to the low frequency of TERT promoter mutations. Cumulative analyses of grade 2 and 3 as well as of primary diagnosed and recurrent lesions might have biased our results but are common due to the low incidence, especially of anaplastic meningiomas. Due to the long inclusion period, the lack of DNA in sufficient quality as well as eligible MRI data increased the number of dropouts (see Supplementary Figure S1). As the WHO classification of brain tumors suggests analyzes for TERT promoter mutations predominantly in lesions with grade 2/3 borderline histology [4], we did not investigate benign meningiomas in our series. Thus, transferability of our findings to meningiomas in general is limited. For statistical reasons, the number of mutant cases in our cohort was artificially increased by sampling several ROIs, while the spatial intratumoral heterogeneity of TERT promotor mutations has been described previously [32]. Finally, the second molecular alteration sufficient for diagnosis of anaplastic meningioma, CDKN2A/B co-deletion, has not been analyzed. Regarding our radiomics machine learning approach, it is important to emphasize that major limitations apply. Our study shows effective classification based on a single-center dataset with the selected features; further studies should aim at evaluating and validating our approach with multi-center data for generalizability and robustness and investigation of our criteria and features and considering incorporating other features, which would improve prediction of TERT promotor mutation [33]. Moreover, it is important to note that standard metrics to evaluate the power of machine-learning-based prediction methods are lacking; therefore, comparison with other prediction models is difficult. Other future challenges that have to be addressed before a clinical application are the standardization of preprocessing of images and the creation of large public datasets for model training and validation [34].

In conclusion, we revealed radiomics to reliably predict TERT promotor mutations in high-grade meningiomas with good to excellent discriminatory performance. Imaging analyzes using radiomics might therefore be a helpful implementation to enable more precise molecular screening investigations, ultimately impacting postoperative therapy.

## Data Availability

The datasets generated during and/or analyzed during the current study are available from the corresponding author on reasonable request.

## Supplementary Materials

The following supporting information can be downloaded at: https://www.mdpi.com/article/10.3390/cancers15174415/s1, Figure S1: Flowchart of patient selection; Table S1: Baseline clinical and neuropathological characteristics of the study cohort; Table S2: TERT promotor mutation status of the training and independent test data; Table S3: Univariate discriminatory power of the final model features.
