# Supplementary material for: Radiomics-Based Prediction of TERT Promotor Mutations in Intracranial High-Grade Meningiomas
Source: Cancers (Basel). 2023 Sep 4;15(17):4415. doi: 10.3390/cancers15174415 (PMC10486806; doi:10.3390/cancers15174415)
Supplement: Supplementary file 1 [file cancers-15-04415-s001.zip › cancers-2553204-supplementary.pdf]

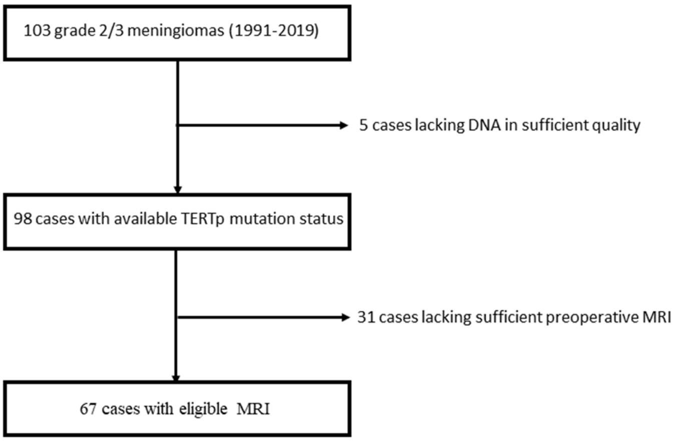

Figure S1. Flowchart of patient selection. All cases who underwent surgery for neuropathologically confirmed grade 2/3 meningioma in our institution were included. However, due to the long inclusion period, DNA as well as eligible MRI was not available in a considerable portion of patients. TERTp = TERT promotor; MRI = magnetic resonance imaging

Table S1. Baseline clinical and neuropathological characteristics of the study cohort.

**Variable**	**N (n%)**
Age (median, range)	66 years, 23-86
Sex	
Males	34 (51%)
Females	33 (49%)
Tumor location	
Convexity	37 (55%)
Falx/ parasagittal	10 (15%)
Skull base	18 (27%)
Others	2 (3%)
Initial diagnosis	39 (58%)
Recurrence	28 (42%)
TERT promotor mutation	9 (14%)
C228T	7
C250T	1
C250 and C228T	1
WHO grade	
2	62 (93%)
3	5 (7%)

Table S2. TERT promotor mutation status of the training and independent test data. For statistical reasons, number of samples with mutations was artificially increased (see Table S1).

	**Training data**	**Independent test data**	**Total data**
**Number of images**	94	23	117
**TERT mutation (in %)**			
Yes (mutated)	43.62	43.48	43.59
No (wildtype)	56.38	56.52	56.41

Table S3. Univariate discriminatory power of the final model features. AUC values for all images and one image. The column shows the mean values obtained after 10 repetitions.

Level of importance	Feature name	AUC: All images used	Mean AUC: One image used per patient
1	MCC	0.760	0.696
2	Location = falx (yes/no)	0.719	0.736
3	DependenceEntropy	0.828	0.807
4	LargeDependenceHighGrayLevelEmphasis	0.828	0.833
5	Minimum	0.699	0.742
6	RunEntropy	0.809	0.723
7	SizeZoneNonUniformity	0.818	0.746
8	GrayLevelNonUniformity.2	0.827	0.789
